# Clinical Outcomes and Quantitative Margin Analysis of a Universal Adhesive Using a Randomized Clinical Trial over Three Years

**DOI:** 10.3390/jcm11236910

**Published:** 2022-11-23

**Authors:** Rainer Haak, Melissa Sophie Werner, Hartmut Schneider, Matthias Häfer, Ellen Schulz-Kornas

**Affiliations:** Department of Cariology, Endodontology and Periodontology, University of Leipzig, Liebigstraße 12, 04103 Leipzig, Germany

**Keywords:** universal adhesive, clinical trial, FDI criteria, quantitative marginal analysis, marginal gap

## Abstract

The effectiveness of a universal adhesive applied in different application modes for the preparation of Class V composite restorations was evaluated both clinically and by quantitative marginal analysis (QMA). In each of the 22 patients, four non-carious cervical lesions (NCCL) were restored with Filtek^™^ Supreme XTE (3M). The adhesive Scotchbond^™^ Universal (SBU, 3M) was applied in self-etch (SE), selective-enamel-etch (SEE) or etch-and-rinse (ER) modes. The etch-and-rinse adhesive OptiBond^™^ FL (OFL, Kerr) served as a control. The restorations were clinically evaluated (FDI criteria) after 14 days (BL), 6, 12, 24, and 36 months. Additionally, QMA was conducted on all restorations of 11 randomly selected patients. The FDI criteria and marginal gap were statistically compared between the groups at each recall as well as for the time periods between recalls. The cumulative failure rate was non-significantly higher in the OFL group when compared to all of the SBU groups. Marginal adaptation in the OFL and SBU-SE/ER groups was significantly decreased (BL-36 m, *p*: 0.004) in comparison to the SBU-SEE group (BL-36 m, *p*: 0.063). More marginal gaps were found in the OFL group than in the SBU-SEE (BL to 36 m, *p*: 0.063–0.003) and SBU-ER (24/36 m, *p*: 0.066/0.005) groups as well as in the SBU-SE group when compared to the SBU-SEE (12–36 m, *p_i_* ≤ 0.016) and SBU-ER (24/36 m, *p*: 0.055/0.001) groups. SBU-SEE performed most effectively. The clinical evaluation and QMA corresponded, yet QMA detected group differences earliest after 6 months and is thus a valuable extension to clinical evaluations.

## 1. Introduction

A reliable bond between tooth structure and restoration material is the prerequisite for the clinical success of a long-lasting restoration [1,2,3,4]. The development of universal adhesives (UA), also known as multi-mode adhesives (MM), has enhanced the development of self-conditioning adhesives (SEA) to a level where they should be able to compete with classic multi-step adhesives [2,5,6,7,8].

Universal adhesives save time by reducing the number of treatment steps. In addition, according to the product manufacturers, universal adhesives are more failure-tolerant than classic adhesive systems due to lower technology sensitivity [9]. The improved bonding properties of UAs display moisture robustness when bonding to both wet and dry dentin, allowing a more reliable bond to enamel or dentin to be established [10]. In addition, UAs can be used in both the self-etch (SE) and etch-and-rinse (ER) application modes as well as for selective-enamel-etch (SEE), offering maximum flexibility to the practitioner according to the clinical situation [11,12]. Additionally, selective enamel etching with phosphoric acid is recommended for UAs [10,13,14,15], thereby reducing the risk of postoperative sensitivity and marginal discoloration (for a systematic review, see [16]).

Most UA systems contain the functional monomer 10-MDP (10-methacryloyloxydecyl dihydrogen phosphate) [17], which ensures efficient conditioning of dental hard tissues for micromechanical bonding as well as effective ionic bonding to hydroxyapatite [10,18]. Numerous in vitro studies have documented that UAs exhibit comparable performance to classic adhesive systems [5,6,10,19,20]. However, in light of the well-known discrepancies that occur when transferring in vitro results to a clinical situation and the conflict between system simplification on the one hand and the desired acceptable clinical results on the other, the possibilities and limitations of universal adhesives can also be viewed critically [21]. Nevertheless, a few short- and medium-term clinical studies have been published in the last decade [10,22,23,24,25].

In a three-year clinical study, composite restorations were placed in all three conditioning modes utilizing Scotchbond™ Universal Adhesive (SBU, 3M Deutschland GmbH, Seefeld, Germany). Restorations in which the three-step etch-and-rinse adhesive OptiBond^™^ FL was applied served as a reference system (OFL, Kerr GmbH, Herzogenrath, Germany). Parallel to the clinical evaluation of the restorations from baseline to 6 months, bond failure at the tooth-composite interface was assessed using optical coherence tomography (OCT) imagery. At 6 months, the restorations placed using the adhesive OFL showed an increased dentin-composite bond failure when compared to those placed using the SBU adhesive, regardless of the chosen conditioning mode of SBU [22]. However, the decreased interface integrity observed in OFL could not be clinically confirmed at 6 months. Nevertheless, following this short observation period, the authors concluded that the subtle interfacial adhesive defects found in the OFL group versus those in the SBU groups might well affect the clinical performance of OFL throughout the study period. In sum, it is still not clear which application modes should be used and when [24,25]. It has rarely been the case that the universal adhesives have been tested in all three modes on the one hand and, according to FDI, against a reference standard on the other. Furthermore, it has been found that different UAs do not necessarily show the same performance in the respective application mode [26]. Therefore, our aim was to investigate the question of the most clinically favorable application mode of the longest established and proven universal adhesive in the challenging scenario of restoring NCCLs and to compare it with a highly established reference adhesive.

In this study, the universal adhesive Scotchbond Universal in all application modes and Optibond FL were further clinically evaluated for a total of three years. Additionally, a quantitative analysis of the restoration margins (QMA) was performed parallel to the clinical evaluation. We aimed to compare the universal adhesive (SBU, three application modes) with OFL (control). The following hypotheses were tested:

1. Both in clinical evaluation (primary outcome) and quantitative marginal analysis (secondary outcome), SBU has an increased performance when compared to that of OFL (adhesive evaluation, application mode, conformity of methods);
2. The marginal gap generally increases with the passing of time (gap progression);
3. Quantitative margin analysis identifies group differences before they become visible within clinical evaluation parameters (power of methods).

## 2. Materials and Methods

### 2.1. Study Design

The Ethics Committee of the University of Leipzig approved the randomized controlled clinical three-year study with reference number 196-14-140420114. The study, as previously described, was registered at the German Clinical Trials Register #DRKS00011084 [22], conducted from 2015 to 2019 and was completed as planned after 36 months. Adult participants were recruited from the Departments of Cariology, Endodontology and Periodontology at the University of Leipzig. The participants, the investigators (clinical assessment (MH) and quantitative margins analysis (MW)), as well as the data evaluator (MW) were blinded to group affiliation. All participants were informed about the study and signed a declaration of consent. Based on a four-arm parallel trial design, four non-carious cervical lesions (NCCL, randomized system allocation) were restored per participant by a calibrated dentist. The calibration of the clinical operator was performed under the supervision of the clinical investigator (MH). This involved the placement of 12 NCCL restorations in vitro and by evaluating the quality of the restorations, in particular the marginal adaptation (specifically to avoid composite excess), with OCT.

### 2.2. Study Population

**Figure 1:**
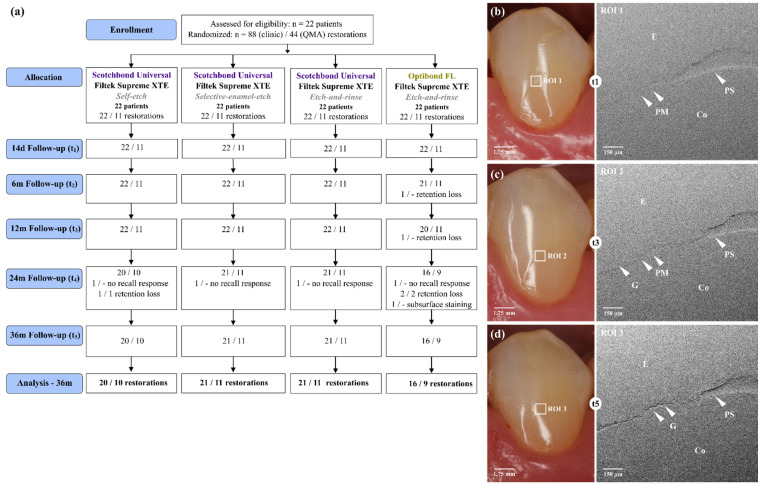
Experimental design and images. (**a**) CONSORT flow diagram. Clinical images at three recalls indicating the restoration and the region of interest (ROI) with areas of composite (Co), enamel (E), and the restoration margin; Sample 08, tooth 35. (**b**) The clinical and SEM image 14 days (t1) after restoration placement. Marginal adaptation and marginal staining were assessed with a score of 1 each. The SEM image shows a perfect restoration margin (PM) and a positive ledge (PL). (**c**) At 12 months (t3), marginal adaptation and marginal staining were clinically scored as 1 each, and a perfect margin, a small marginal gap (G) and a positive ledge appear in the SEM image. (**d**) At 36 months (t5), marginal adaptation and marginal staining were clinically scored as 1 each again, with the SEM image showing a more extended marginal gap and a positive ledge.

The current clinical study included 22 participants with four non-carious cervical lesions (NCCL) each, located in the incisors, canines and premolars (Table 1, Figure 1a). The lesions of each participant were allocated to the intervention groups according to a computer-generated list of random numbers, which was created by an independent member of the dental clinic not involved in this trial. Eleven of the 22 patients were randomly selected for the quantitative marginal analysis via a block of 4 randomizations (Table 1). The random selection of restorations for quantitative margin evaluation was performed after 36 months.

The determination of sample size was based on a preliminary study by the authors [27] comparing clinical restoration assessment in 19 patients and quantitative analysis of restoration margins in nine of the 19 patients with nine pairs of restorations (replica pairs) per study group. Significant group differences were shown clinically (cumulative failure marginal integrity) and based on QMA (marginal gap difference in the groups) using these group sizes. Sample size calculation for QMA based on the group differences found in the study [27] for the marginal gap of 28.4% resulted in a test power of 80% already for n = 10 pairs of restorations (α = 0.05; PS-Power and Sample Size Calculation, version 3.0.43, Vanderbilt Univ., Nashville, TN). The sample size n = 22 for the clinical evaluation resulted from the number of patients available in the study.

Participants were included if they were 18 years of age or older, the trial teeth had a relationship with a natural antagonist, and the trial teeth tested positive for sensitivity (CO_2_ snow). Furthermore, participants were required to have complete dentition (≥20 teeth), and the trial teeth had no contact with any other restoration. The following exclusion criteria were applied: pregnancy, alcohol or drug dependency, wear facets on the study teeth, material allergies according to the patient’s information, as well as impossible contamination control and a probing depth >4 mm. After the start of the trial, there were no changes in the inclusion and exclusion criteria of the patients or in methodological aspects such as restoration procedure and restoration evaluation.

### 2.3. Restorative Procedure

The universal adhesive Scotchbond^™^ Universal (3M Deutschland GmbH, Seefeld, Germany) was applied in combination with the composite Filtek^™^ Supreme XTE (3M Deutschland GmbH, Seefeld, Germany) in the conditioning modes of self-etch, selective-enamel-etch and etch-and-rinse. The OptiBond^™^ FL adhesive system (Kerr GmbH, Herzogenrath, Germany) served as a reference system (ER mode; Table 2). Restorations were performed on 22 patients, each with 4 non-carious cervical lesions on the incisors, canines, or premolars (88 lesions in total). The randomized allocation of the teeth and lesions in the study groups is shown in Table 1 and Table 2 and Figure 1a. Lesion sizes were classified and categorized as shallow (depth ≤ 1 mm), medium (depth ≤ 2 mm) or deep (depth > 2 mm) before restoration. Tooth wear was determined according to the Smith and Knight Tooth Wear Index [28] and took into account teeth with a tooth wear index score between 2 and 4. All restorations were placed according to Table 2 and the previously described protocol [29].

### 2.4. Manufacture of the Impression

All restorations were cleaned with a soft rotating brush and dried with oil-free compressed air prior to taking impressions. The impressions were made with polyvinylsiloxane A-silicone Aquasil Ultra LV (Dentsply Sirona Deutschland GmbH, Konstanz, Germany). The material application was initially conducted with a flow direction via sulcus until sufficient stability of the impression was achieved. The impressions were subsequently removed, disinfected, and stabilized with Aquasil Soft putty (Dentsply Sirona Deutschland GmbH, Konstanz, Germany).

### 2.5. Replica Production and Mounting for SEM Imaging

The replicas were fabricated in the clinic’s research laboratory. They were made of epoxy resin (Stycast 1266; Emerson & Cuming, Westerlo, Belgium), trimmed for mounting on SEM specimen holders (sample plate with pen, 12 mm, Plano GmbH, Wetzlar, Germany) using carbon (Leit-C-Plast, Neubauer Chemicals, Münster, Germany), and sputter coated with gold (10 nm, LOT MiniSputterCoater Automatic MSC1T, Liebscher GmbH, Schöffengrund, Germany).

### 2.6. Study Outcomes

#### 2.6.1. Clinical Assessment

The clinical assessment of the restorations and the fabrication of their impressions were always performed at the same dental chair by the primary examiner (MH). The primary examiner assessed all restored study teeth according to FDI criteria at 14 days (baseline, t1) and thereafter at 6 (t2), 12 (t3), 24 (t4), and 36 months (t5). Photographs of the teeth were taken before and after restoration [29] as well as at the examination times specified above [30].

Dental magnifying glasses (2.5×) combined with explorers (Kit-EX: tip diameter 150 mm, 250 mm; Deppeler SA, Rolle, Switzerland) were used to evaluate the functional, aesthetic, and biological criteria. The sensitivity of the study teeth was checked using CO_2_ snow. A visual analog scale and a periodontal probe were used to check the periodontal status and the probing depth (P15/11.5B6; Hu-Friedy Mfg. B.V., Rotterdam, Netherlands). The criteria were rated as follows: 1 (very good), 2 (good, very good after correction), 3 (sufficient/satisfactory, minor defects), 4 (unsatisfactory but repairable), 5 (poor, replacement necessary) [31].

If the marginal adaptation (MA) was not rated with a score of 1 in the first 14 days after restoration, individual minor marginal fractures were removed until the required MA of very good (score 1) was achieved. Restorations that were assessed as clinically unacceptable in 1 criterion were excluded from the study and replaced or repaired. The criteria defining the clinical endpoint were as follows: fracture and retention, marginal adaptation (MA), and marginal staining (MS) [29].

#### 2.6.2. Quantitative Margin Analysis

The replicas were imaged by the investigator (MW) using a scanning electron microscope of the clinic (Phenom G2 Pro, Phenom-World BV, Eindhoven, NL; 5 kV, 100–200× magnification) at t1 (14 days), t2 (6 months), t3 (12 months), t4 (24 months) and t5 (36 months).

The following parameters were used for the margin analysis: perfect margin (PM), positive ledge (PL), negative ledge (NL), gap (G), margin irregularity (MI), and artifact (A). The percentage amount of gap parameters in relation to the total length (arithmetic means) at each examination time was determined for each group according to these parameters. The data evaluator (MW) was calibrated and trained by an experienced operator. Fiji/ImageJ version 1.48f and plugin QuantiGap [32] were used for margin analysis. The sum of the parameters positive ledge, negative ledge, margin irregularity, and perfect margin resulted in the main characteristic of “no gap,” which is given as a percentage of the total length (without artifact length). The parameter G was used for further statistical analysis.

### 2.7. Statistical Analysis

#### 2.7.1. Clinical Assessment

The clinical assessment was carried out as previously described [22]. The retention rates were calculated at the time of each examination as follows: failure percentage = [(F_previous_ + F_current_)/(F_previous_ + N_current_)] × 100, %; whereby F_previous_ represents the number of previous failures before the current recall examination, F_current_ and N_current_ represent the number of failures and the number of restorations seen in the current recall. At the examination times, cumulative failure rates (CFR) were determined for each criterion and for the sum of all criteria (confidence intervals, Clopper-Pearson).

SPSS Statistics for Windows 23.0 (IBM Corp. Armonk, NY, United States of America) was used to conduct the statistical analyses. All parameters between groups per examination time (horizontal testing) and within each group over the relevant time intervals (vertical testing) were compared using the Mc-Nemar test (2-sided, α = 0.05). Due to the exploratory nature of this study, *p*-values were reported as raw values, and no adjustment for multiple testing was made.

#### 2.7.2. Quantitative Margin Analysis

If a restoration was lost, the missing value was replaced by the highest value of the respective group at the time of loss (missing data imputation). The normal distribution was validated according to the Shapiro–Wilk and Kolmogorov–Smirnov tests. The Shapiro–Wilk test revealed a deviation from a normal distribution for most data. Therefore, non-parametric tests were used. Friedman’s and Wilcoxon’s tests were used to compare the groups at each recall time as well as to make comparisons within the groups over a 36-month period (dependent samples). Raw *p*-values were once again reported in order to compare the groups and the values within the groups over time (significance level α = 0.05).

Five replicates were randomly selected for an interpersonal comparison and evaluated by 2 raters prior to stating any further data evaluation. The determination of the marginal gap criterion showed 99.8% agreement. The characteristic “no gap” was calculated based on the sum of the individual lengths of the positive and negative ledge, margin irregularity, and perfect margin. There was an interpersonal difference between rater 1 and rater 2 within the positive step and a marginal irregularity of 12.8%. However, since these characteristics were added to the negative step and perfect margin and resulted in the characteristic “no gap,” an interpersonal agreement on the characteristics gap and no gap was achieved (5 comparisons, Wilcoxon test, no significant difference *p* = 1.000). The average standard errors were 4.9–3.9% (“no gap”).

## 3. Results

### 3.1. Clinical Assessment

Seventy-eight of the 88 restorations were evaluated at 36 months. The reassessment rate within the groups varied from 72.7% to 95.5% (Table 3). The differences in clinical criteria between the groups at the time of each recall (horizontal testing) and per group over the entire assessment period of 36 months (vertical testing), as well as the cumulative failure rates with confidence intervals (24 m and 36 m), are documented in Table 4, Table 5 and Table 6.

All restorations in the SBU-SEE and SBU-ER groups were in situ after 36 months. One restoration in the SBU-SE group was lost after 24 months. In the OFL group, five restorations failed over the 36-month period: one loss at 6 and one at 12 months, two losses at 24 months, and one due to subsurface staining at 24 months.

There were no significant group differences in the clinical criteria within the study period (Table 4). Nevertheless, OFL exhibited a trend towards increased marginal staining (MS, score 2) and decreased marginal adaptation (MA, score 2) at 36 and 6 months, respectively, when compared to the SBU-SEE group. The cumulative failure rate was also not significantly higher in the OFL group when compared to that of all the SBU groups, with a trend towards the 24 m and 36 m periods and the SBU-SEE vs. OFL and the SBU-ER vs. OFL comparisons (*p_i_* = 0.063, Table 5). Therefore, differences between groups were presented descriptively using confidence intervals (24 m, 36 m) (Table 5).

The restorations in all groups showed progressive marginal deterioration from scores of 1 to 2 within the clinical acceptance range (Table 4), with a significant shift in marginal staining in the SBU-ER and OFL groups and in marginal adaptation in the SBU-SE/ER and OFL groups from BL to 36 months (Table 6). On the other hand, the SBU-SEE group showed a trend toward marginal adaptation (Table 6).

### 3.2. Quantitative Margin Analysis

**Figure 2:**
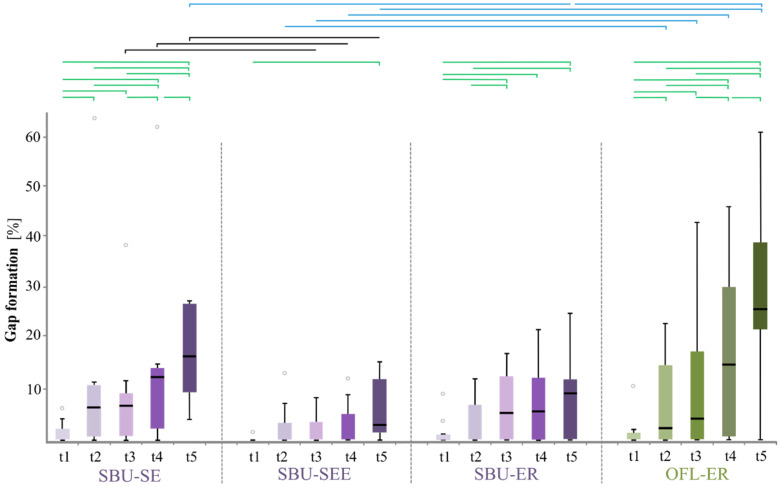
Boxplots of mean marginal gap formation (%) on restorations placed with composite and Scotchbond Universal in self-etch (SBU-SE), selective-enamel-etch (SBU-SEE) and etch-and-rinse (SBU-ER) application modes or with OptiBond FL (OFL) for the day 14 (t1) and 6 (t2), 12 (t3), 24 (t4) and 36 months (t5) follow-ups. Significant group differences (*p_i_* < 0.05) at each follow-up (black, blue) and the significant increase in marginal gap in the groups over time (green) are marked.

Marginal gap formation was found in all groups and at all follow-up examinations (Table 7, Figure 1b–d and Figure 2). Group differences at each recall (horizontal testing) were markedly group-specific and partially detected at 6 months. The same is true for the marginal gap increase over the 36-month period (vertical testing, Figure 2). The replica technique made it impossible to discriminate between enamel, dentin, and cement margins. The gap formation was mainly detected in the cervical parts of the margin.

Scotchbond Universal (all modes): The lowest marginal gap formation invariably occurred in the SEE mode, followed by the ER and SE modes (Table 7, Figure 2). The group differences were significant between the SEE and SE groups from 12 m to 36 m and at 12 m between the SEE and ER groups. Regarding the SE and ER modes within the SBU group, tendentially fewer gaps appeared in the ER mode than in the SE mode from 24 m (Table 7). As shown in Figure 2 and Table 8, a significant progression of the marginal gaps was observed over time, both between consecutive time points and extended periods. The gaps also generally increased in the SE mode, however, between 6 m and 12 m. The trend was limited to the more extended period BL-36 m in ER mode. In ER mode, this effect started at 6 m (BL trend), while in SEE mode, it was limited to the longer BL-36 m period (BL-12/24 m trends, Table 8).

OptiBond FL: More marginal gap was observed in the OFL group than in the SBU groups at all points in time. When compared to the SBU-SEE group, this was statistically significant from 6 m (BL trend, Table 7) to 36 m, and when compared to the ER mode group, a statistical significance was found at the 36 m time point (24 m trend). By contrast, the differences between the OFL group and the SBU-SE mode group were never significant. Over time, with the exception of the 6 m–12 m period (trend), the gap increase was always significant between all consecutive time points as well as over large intervals (Figure 2, Table 8).

### 3.3. Clinic and QMA

The clinical assessment of restoration margin quality, especially marginal adaptation, and the quantification of the restoration margin gap (QMA), its analytical equivalent, were conformal. Both assessments revealed higher marginal quality in the SBU-SEE group when compared to the SBU-SE and SBU-ER mode groups as well as to the OFL group. Table 6 and Table 8 show that this was also true for the frequencies of clinical score 2 (especially for marginal adaptation), marginal gap (QMA), and gap progression. The non-significantly increased clinical retention losses and, according to a trend, higher cumulative failure rates in the OFL group when compared to all SBU groups (from the 6-month recall) correspond to the significant or partially non-significant increases in marginal gap formation (QMA) from BL.

Compared to the quantitative marginal analysis, there is a considerable time lag when detecting significant group differences via clinical assessment (more than 36 m, cumulative failure rate). When using QMA, a significant group difference in the performance of adhesives was detected as early as 6 months (SBU-SEE/OFL) or 36 months (SBU-ER/OFL) after restoration placement. The difference between the SBU-SEE and OFL groups was reproduced at 12, 24, and 36 months, with increased marginal gap formation and further retention loss in the OFL group. While the group differences in marginal gap revealed with QMA are largely not reflected clinically in the MS and MA criteria (Table 4), the decrease in marginal clinical quality (MA, score 1–2, Table 6) correlates with the increase in the measured marginal gap (Table 8).

## 4. Discussion

Parallel to the clinical evaluation in the current study, the adhesive Scotchbond Universal was evaluated by quantitative SEM analysis. Using this combination of methods to assess adhesives proved to be very meaningful [20,22,27,29]. Clinical evaluations usually only allow statements regarding the performance of restoration systems after extended study periods [33,34,35,36,37]. In this study, by contrast, a hypothesis on the performance of the SBU (all application modes) and OFL adhesives was formulated a mere 6 months after the placement of the restorations, based on a tomographic evaluation of the tooth-composite bond failure and a parallel clinical evaluation [22]. The combination of a clinical evaluation and QMA incorporates the following two advantages of QMA: First, it is highly standardized and has become a standard for margin analysis over the past 30 years [3,38,39,40,41], and second, its detailed examination of the margins makes it more sensitive to early gap detection [39,42,43,44,45].

Scotchbond Universal adhesive has an increased performance when compared to the reference system OptiBond FL. Non-significant or trending lower cumulative failure rates were obtained with SBU in all three application modes from 6 to 36 months. This became more evident through quantitative margin analysis as the differences in the quality of the restoration margins between the systems or groups could also be partially validated statistically. When comparing the SBU application modes, QMA, analogous to clinical analysis, showed maximum performance in SEE mode, followed by the ER and SE modes. Thus, clinical evaluation and QMA are substantially conforming, and hypothesis 1 can be accepted. Additionally, higher clinical progression in retention loss is evident in the OFL group when compared to the SBU-SE group over 36 months (Table 3, Figure 2). Nevertheless, no significant group difference between OFL and SBU-SE was detected by QMA at any time (Table 7, Figure 2).

Our results are in line with the conclusion of this trial’s 6-month optical coherence tomography (OCT) evaluation [22]. The OCT evaluation of the NCCLs for the first 6 months showed that more failures occurred at the enamel-composite interfaces in the SE mode than in the SEE and ER modes. By contrast, at the dominant dentin-composite interface, the lowest interfacial gap formations were measured in the modes ER and SEE, and a smaller interfacial gap was measured in all SBU modes than in OFL. Clinically, however, no inferior performance of OFL was observed. Nevertheless, the authors concluded from the subtle interfacial adhesive defects at the dentin-composite interface in the OFL group that this could affect clinical outcomes in the further course of the study. This conclusion was clinically confirmed in this study after 36 months and previously validated by QMA at earlier time points. In a meta-analysis [45], the authors showed that SBU on enamel achieves a higher bond strength in the ER mode than in the SE mode. In a previous study, SBU was found to rapidly form gaps in the enamel when used as a mild SE adhesive (pH 2.7) without phosphoric acid etching of the enamel [46]. These studies support the results presented here, where the SBU with selective enamel etching or in etch-and-rinse mode showed the highest proportion of gap-free restoration margins both clinically and in QMA.

The occurrence of marginal gaps is the morphological correlate of poor micro-retentive or chemical bonding between the tooth substrate and the composite restoration. Gap progression is expected to result from complex processes of physical and chemical degradation of the tooth-composite bonding zone in the course of clinical loading of restorations. The penetration of bacteria into the marginal gaps and their metabolic products also contributes to a loss of integrity [47]. A progression of marginal gap formation was observed in all groups with the passing of time (hypothesis 2). This effect was most noticeable in the OFL group.

The quantitative margin analysis has high statistical power [27,48,49]. It thus allows for earlier statements on the performance of composite restorations and reveals group differences before they become visible in a clinical evaluation. This was also observed in the current study, although only half of the clinically examined restorations had to be assessed with QMA on account of there being 11 restorations per group. The presented results reconfirm that the quantitative determination of higher resolved gaps (SEM) allows for an earlier statement regarding the marginal adaptation of the restorations prior to defects becoming clinically visible. Consequently, hypothesis 3 can also be accepted.

This study’s results align with several prospective clinical studies regarding the clinical performance of SBU. It was applied to NCCLs and indicated acceptable clinical outcomes after 6 [50], 18 [19], and 36 months [51]. Nevertheless, the weaker performance of OFL when compared to SBU was initially surprising as OptiBond FL is a widely used reference system but is critically seen [52,53,54,55,56]. An indication of the possible superiority of SBU in the ER and SE modes over a classical etch-and-rinse adhesive was given in a 24-month in vivo study [57]. Previous clinical studies on NCCLs have shown that failure rates in the OptiBond FL groups ranged widely: from 0% [52] to an accepTable 4% after one year [53], 9% after 5 years [58], yet also 16.7% at 12 months [23]. In the current study, however, 20% of OFL restorations were lost after 24 and 36 months. To our knowledge, the composition of the batches of OptiBond FL used in the present study compared to those in previous studies was changed, and the adhesive was applied according to the manufacturer’s instructions [24]. The operator (PS) in this study had many years of professional experience with OptiBond FL and was extensively calibrated prior to the study. Thus, these factors can be ruled out as reasons for OFL’s inferior performance. The high number of restoration losses may be due to insufficiently pronounced micro-retention patterns on the tooth substrate. As already suggested by others [59], a mechanical roughening with fine-grain diamonds could contribute to an improved bond when using OFL and result in the increased retention of the restorations. Yet, this is still a controversial topic in the literature ([60,61]). More extensive chemical conditioning with phosphoric acid gel would have the same result. In contrast to the freshly prepared lesions of an in vitro study [62], the conditioning of NCCLs is compromised by hypermineralization of sclerotic dentin, which hinders monomer penetration [63]. On the other hand, Peumans et al. [64] prepared the NCCL by beveling the enamel to a depth of 1–2 mm and leaving the dentin walls mechanically de-roughened. Finally, the adhesive’s technical sensitivity must be considered a performance-determining factor. For example, the number of steps necessary when using a multi-step adhesive such as OFL can lead to more application errors. The extent of the boxplots in the diagrams of Figure 2, which results from the scatter of the measured values of the marginal gap in the groups, can be seen as a measure of the technique sensitivity of the adhesives [29]. Accordingly, it is evident that at baseline, the lowest spread of values was present in the SEE group, while at the 36-month follow-up, the most considerable dispersion of values was in the OFL group. As early as 6 months, the boxplots in the OFL group were increasingly more extended than in the SBU groups, which is consistent with the results of the clinical examination and the marginal analysis.

A further argument for the enhanced SBU performance is the universal adhesive’s main components. Like most universal adhesives, SBU contains 10-MDP (methacryloyloxydecyl dihydrogen phosphate). 10-MDP forms stable crystals with the calcium ions of the incompletely dissolved hydroxyapatite. This chemical binding behavior reduced nanoleakage after 6 months [65], and the additional chemical binding component may have also contributed to the lower retention losses of the SBU groups in the present study. In addition, there is little evidence of chemical interaction of GPDM in OFL [66,67], and thus SBU can be expected to form a more stable adhesive-dentin bond with the sclerotic dentin of an NCCL [68]. According to the data available so far, the marginal gap formations and retention losses are clearly interrelated in the respective groups.

Eleven restorations per group were included in the QMA. The basic limitation of such studies with a high effort of measurement methodology is that possibly small group differences cannot be statistically validated. In order to ensure the test power of 80%, n = 11 restorations were included in the study instead of the calculated 10. The patients/replicas included had 36 months of continuous recall in order to fully reflect the progression of the margin for the selected restorations over time. Therefore, the random selection of restorations for quantitative margin evaluation was performed after 36 months. Thus, the study reconfirmed that QMA on composite restorations has higher statistical power than a clinical assessment based on fractures/retention, marginal adaptation and marginal staining [27,48,49]. This means that considerably larger sample sizes and/or longer study periods are required for clinical evaluation to statistically validate group differences. For example, in previous clinical studies on OFL that were based on 55 [29] and 50 restorations [23], the authors were able to demonstrate significantly increased cumulative failure rates per group when compared to SBU (SE mode) and iBond Universal (SE mode) after 12 months. However, this was not detectable with smaller sample sizes of 22 or 29 restorations.

As a non-invasive method, quantitative marginal analysis allows multiple quantifications of marginal parameters in vivo, providing relevant information on the clinical performance of adhesives or restorative systems after only 6 to 12 months, even with small sample sizes. If the methodological prerequisites are in place, the advantages outweigh the disadvantages, for example, replica preparation, SEM imaging and image evaluation of the additional methodological effort. However, one must bear in mind that the associated margin evaluation is merely morphological and does not include additional biological, esthetic, and functional parameters that may be required and useful for a comprehensive restoration evaluation [30,31].

## 5. Conclusions

The application of Scotchbond Universal in the SEE and ER modes resulted in the highest performance throughout the evaluation period, both clinically and in terms of the formation of restoration margin gaps (QMA), with the SEE mode prevailing. However, predictions for individual restorations are not possible, as even clinically acceptable restorations sometimes showed increased gap formation yet were still in situ after 36 months. The quantitative marginal analysis provides earlier information on adhesive performance, even with small sample sizes.

## Figures and Tables

**Table 1:** Study groups and selection of teeth and lesions (adapted from [22]).

Group	SBU-SE	SBU-SEE	SBU-ER	OFL-ER
N_Clinic_/N_QMA_	22/11	22/11	22/11	22/11
Adhesive	Scotchbond Universal (SBU)			OptiBond FL (OFL)
Application mode	self-etch (SE)	selective-enamel-etch (SEE)	etch-and-rinse (ER)	etch-and-rinse (ER)
Composite	nanocomposite Filtek^™^ Supreme XTE (3M)			
Lost restorations	1 ^1^	-	-	5 ^2^
Location				
maxilla	10/4	10/5	14/7	10/6
mandible	12/7	12/6	8/4	12/5
Lesion borderline				
enamel	-/-	-/-	-/-	-/-
dentin	2/-	3/1	2/2	4/2
mixed (enamel/dentin)	20/11	19/10	20/9	18/9
Lesion depth				
shallow (<1 mm deep)	-/-	1/-	-/-	2/1
medium (1–2 mm deep)	22/11	21/11	21/11	19/10
deep (>2 mm)	-/-	-/-	1/-	1/-

^1^ retention loss; ^2^ retention loss (4×) plus subsurface staining (1×).

**Table 2:** The adhesive system, composition and procedure of application according to the manufacturer’s recommendations.

Material	Composition	Self-Etch Mode	Selective-Enamel-Etch Mode	Etch-and-Rinse Mode
Scotchbond Universal Etchant ^a^	35% phosphoric acid		1. Apply etchant for 30 s on the enamel. 2. Rinse with water for 20 s and dry with water- and oil-free air	
Scotchbond Universal ^a^	10-MDP, HEMA, silane, dimethacrylate resins, Vitrebond^™^ copolymer, filler, ethanol, water, initiators (LOT 552577)	1. Actively apply the adhesive to the cavity for 20 s. 2. Gently air-dry the adhesive for approximately 5 s for the solvent to evaporate. 3. Light cure for 10 s (>1000 mW/cm^2^) ^1^.		
OptiBond FL ^b^	FL primer: HEMA, GPDM, MMEP, water, ethanol, photoinitiator (CQ), BHT FL adhesive: Bis-GMA, HEMA, GPDM, GDMA, photoinitiator (CQ), ODMAB, fillers, barium aluminoborosilicate (LOT 4964258)			1. Apply etchant for 15 s (dentin 15 s, enamel 30 s). 2. Rinse thoroughly for 15 s; air dry for 3 s (do not overdry). 3. Actively apply the primer for 15 s; air dry for 5 s. 4. Apply adhesive with a light brushing motion for 15 s; air thin for 3 s; light cure for 20 s (>1000 mW/cm^2^) ^1^.
Filtek Supreme XTE ^a^	Bis-GMA, UDMA, TEGDMA, Bis-EMA, silanated silica, silanated zirconia, photoinitiators (LOT 552577)		1. Place restorative in increments. 2. Light cure restorative in increments (body, enamel shades 2.0 mm, 20 s. dentin shades 1.5 mm, 40 s, >1000 mW/cm^2^) ^1^.	

10-MDP = methacryloyloxydecyl dihydrogen phosphate, Bis-GMA = bisphenol A diglycidyl methacrylate; bis-EMA(6)1 = (bisphenol A polyethylene glycol diether dimethacrylate); BHT = butylhydroxytoluene; CQ = camphorquinone; DMA = dimethacrylates; GDMA = glycerol dimethacrylate; GPDM = glycerol phosphate dimethacrylate; HEMA = 2-hydroxyethyl methacrylate, MEHQ = 4-methoxyphenol mono(2-methacryloyloxy) ethyl phthalate; ODMAB = 2-(ethylhexyl)-4-(dimethylamino)benzoate; TEGDMA = triethyleneglycol-dimethacrylate. ^1^ Regular curing light check with curing radiometer (Demetron Model 100, Demetron Res. Corp., Danbury, CT, USA). ^a^ 3M Deutschland GmbH, Seefeld, Germany; ^b^ Kerr GmbH, Herzogenrath, Germany.

**Table 3:** Clinical quality of the restorations from baseline (BL) to 36 months (m); clinical data from a former study [22] of BL/6m are indicated by grey shading.

	SBU-SE					SBU-SEE					SBU-ER					OFL				
	BL	6 m	12 m	24 m	36 m	BL	6 m	12 m	24 m	36 m	BL	6 m	12 m	24 m	36 m	BL	6 m	12 m	24 m	36 m
Restorations assessed, n	22	22	22	21	20	22	22	22	21	21	22	22	22	21	21	22	22	21	19	16
Reassessment rate, %	100	100	100	95.5	90.9	100	100	100	95.5	95.5	100	100	100	95.5	95.5	100	100	95.5	86.4	72.7
Aesthetic Criteria ^1^																				
Non-acceptable, %	0.0	0.0	0.0	0.0	0.0	0.0	0.0	0.0	0.0	0.0	0.0	0.0	0.0	0.0	0.0	0.0	0.0	0.0	5.9 ^3^	5.9 ^3^
Functional Criteria ^1^																				
Non-acceptable, %	0.0	0.0	0.0	4.8 ^4^	4.8 ^4^	0.0	0.0	0.0	0.0	0.0	0.0	0.0	0.0	0.0	0.0	0.0	4.5 ^4^	9.1 ^4^	19.0 ^4^	20.0 ^4^
Biological Criteria ^1^																				
Non-acceptable, %	0.0	0.0	0.0	0.0	0.0	0.0	0.0	0.0	0.0	0.0	0.0	0.0	0.0	0.0	0.0	0.0	0.0	0.0	0.0	0.0
Cumulative Failure Rate (Total Score) ^2^																				
Non-acceptable, %	0.0	0.0	0.0	4.8 ^4^	4.8 ^4^	0.0	0.0	0.0	0.0	0.0	0.0	0.0	0.0	0.0	0.0	0.0	4.5 ^4^	9.1 ^4^	23.8 ^5^	23.8 ^5^

^1^ cumulative over time, ^2^ cumulative all criteria, ^3^ caused by subsurface staining, ^4^ caused by retention loss, ^5^ retention loss plus subsurface staining.

**Table 4:** Marginal staining, marginal adaptation (score 2, %) and fractures/retention (score 5, %). Group differences (*p_i_*) from baseline (BL) up to 36 months (m); clinical data (verbal description only) from a former study [22] of BL/6m are indicated by grey shading.

Groups		Marginal Staining ^1^ (Score 2)					Marginal Adaptation ^2^ (Score 2)					Fractures/Retention ^3^ (Score 5)				
Time	BL		6 m	12 m	24 m	36 m	BL	6 m	12 m	24 m	36 m	BL	6 m	12 m	24 m	36 m
SBU SE vs. SEE	% *p_i_*	13.6/4.5 0.5	13.6/4.5 0.625	18.2/4.5 0.375	25/9.5 0.375	30/19 0.687	0/0 n.c.	22.7/4.5 0.219	36.4/18.2 0.344	35/23.8 0.687	45/23.8 0.344	0/0 n.c.	0/0 n. c.	0/0 n. c.	4.8/0 1.0	4.8/0 1.0
SBU SE vs.-ER	% *p_i_*	13.6/0 0.25	13.6/0 0.25	18.2/9.1 0.625	25/4.8 0.125	30/33.3 1.0	0/0 n.c.	22.7/13 0.687	36.4/31.2 1.0	35/28.6 1.0	45/42.9 1.0	0/0 n.c	0/0 n.c.	0/0 n.c.	4.8/0 1.0	4.8/0 1.0
SBU-SE vs. OFL	% *p_i_*	13.6/4.5 0.5	13.6 /19 1.0	18.2/ 25 1.0	25/29.4 1.0	30/41.2 0.687	0/0 n.c.	22.7/28.6 1.0	36.4/35 1.0	35/37.5 1.0	45/56.3 0.453	0/0 n.c.	0/4.5 1.0	0/9.1 0.5	4.8/20 0.375	4.8/20 0.375
SBU-SEE vs. ER	% *p_i_*	4.5/0 1.0	4.5/0 1.0	4.5/9.1 1.0	9.5/4.8 1.0	19./33.3 0.375	0/0 n.c.	4.5/13.6 0.625	18.2/31.2 0.549	23.8/28.6 1.0	23.8/42.9 0.219	0/0 n.c.	0/0 n.c.	0/0 n.c.	0/0 n.c.	0/0 n.c.
SBU-SEE vs. OFL	% *p_i_*	4.5/4.5 1.0	4.5/19 0.25	4.5/25 0.125	9.5/29.4 0.25	19/41.2 0.063 ^4^	0/0 n.c.	4.5/28.6 0.063 ^4^	18.2/35 0.375	23.8/37.5 0.453	23.8/56.3 0.07	0/0 n.c.	0/4.5 1.0	0/9.1 0.5	0/20 0.125	0/20 0.125
SBU-ER vs. OFL	% *p_i_*	0/4.5 1.0	0/19.0 0.125	9.1/25 0.375	4.8/29.4 0.219	33.3/41.2 1.0	0/0 n.c.	13.6/28.6 0.375	31.2/35 1.0	28.6/37.5 0.727	42.9/56.3 0.219	0/0 n.c.	0/4.5 1.0	0/9.1 0.5	0/20 0.125	0/20 0.125

^1^ In group SBU, no scores ≥3 were detected. In group OFL, one restoration was excluded because of score 5 (see Table 3). All other restorations of the OFL group were rated 1/2. ^2^ In all groups, no scores ≥ 3 were detected. ^3^ retention loss. n.c.: not calculable (McNemar, values are not dichotomous because there is no failure), ^4^ trend.

**Table 5:** Cumulative failure rate (CFR) and confidence intervals (method: Clopper-Pearson).

Groups	Time	CFR, %		Confidence Interval (Prevalence)	
SBU-SE ^3^	24 m	4.8		0.001–0.238 (0.048)	
	36 m		4.8		0.0013–0.249 (0.050)
SBU-SEE ^1^	24 m	0.0		0.000–0.161 (0.000)	
	36 m		0.0		0.000–0.161 (0.000)
SBU-ER ^2^	24 m	0.0		0.000–0.161 (0.000)	
	36 m		0.0		0.000–0.161 (0.000)
OFL ^1,2,3^	24 m	23.8		0.092–0.512 (0.263)	
	36 m		23.8		0.110–0.587 (0.313)

Group differences with CRF: ^1^ SBU/SEE vs. OFL, 24 m and 36 m: *p_i_* = 0.063; ^2^ SBU/ER vs. OFL, 24 m and 36 m: *p_i_* = 0.063; ^3^ SBU/SE vs. OFL, 24 m and 36 m: *p_i_* = 0.219.

**Table 6:** Changes (*p_i_*) in marginal staining (MS), marginal adaptation (MA, score 1 to 2) and fractures/retention (score 1 to 5) per group from baseline (BL) up to 6, 12, 24 and 36 months.

**Marginal Staining Score 2 ^1^**										
	BL-6 m	6–12 m	12–24 m	24–36 m	BL-12 m	BL-24 m	BL-36 m	6–24 m	6–36 m	12–36 m
SBU-SE	1.000	1.000	0.625	1.000	1.000	0.687	0.453	0.625	0.375	0.375
SBU-SEE	1.000	1.000	1.000	0.625	1.000	1.000	0.375	1.000	0.375	0.250
SBU-ER	n.c.	0.500	1.000	**0.031**	0.500	1.000	**0.016**	1.000	**0.016**	0.125
OFL	0.250	0.687	1.000	0.625	0.219	0.219	**0.031**	0.625	0.219	0.250
**Marginal Adaptation Score 2 ^1^**										
	BL-6 m	6–12 m	12–24 m	24–36 m	BL-12 m	BL-24 m	BL-36 m	6–24 m	6–36 m	12–36 m
SBU-SE	0.063 *	0.453	1.000	0.687	**0.008**	**0.016**	**0.004**	0.687	0.289	0.625
SBU-SEE	1.000	0.375	1.000	1.000	0.125	0.063 *	0.063 *	0.125	0.219	1.000
SBU-ER	0.250	0.125	1.000	0.375	**0.016**	**0.031**	**0.004**	0.375	**0.031**	0.250
OFL	**0.031**	0.625	1.000	0.375	**0.016**	**0.031**	**0.004**	0.500	0.063 *	0.125
**Fractures/Retention Score 5 ^2^**										
	BL-6 m	6–12 m	12–24 m	24–36 m	BL-12 m	BL-24 m	BL-36 m	6–24 m	6–36 m	12–36 m
SBU-SE	n.c.	n.c.	1.000	1.000	n.c.	1.000	1.000	1.000	1.000	1.000
SBU-SEE	n.c.									
SBU-ER	n.c.									
OFL	1.000	1.000	0.500	1.000	0.500	0.125	0.125	0.250	0.250	0.500

Bold: significant difference, * trend. ^1^ In group SBU, no scores >2 were detected. In group OFL, one restoration was excluded because of a score of 5 (see Table 3); ^2^ retention loss, representing the cumulative failure rate; n.c.: not calculable, McNemar, values are not dichotomous because there is no failure.

**Table 7:** Mean of marginal gap (%) and group differences at a time (*p_i_*).

Time	SBU-SE vs. SBU-SEE		SBU-SE vs. SBU-ER		SBU-SE vs. OFL		SBU-SEE vs. SBU-ER		SBU-SEE vs. OFL		SBU-ER vs. OFL	
	gap, %	*p_i_*	%	*p_i_*	%	*p_i_*	%	*p_i_*	%	*p_i_*	%	*p_i_*
BL	1.4/0.1	0.125	1.4/1.3	0.844	1.4/2.9	0.831	0.1/1.3	0.375	0.1/2.9	0.063 ^1^	1.3/2.9	0.469
6 m	9.2/2.4	0.156	9.2/3.5	0.156	9.2/9.7	0.627	2.4/3.5	0.625	2.4/9.7	**0.023**	3.5/9.7	0.223
12 m	9.6/1.9	**0.016**	9.6/6.0	0.820	9.6/14.6	0.557	1.9/6.0	**0.039**	1.9/14.6	**0.027**	6.0/14.6	0.275
24 m	12.2/3.2	**0.016**	12.2/7.4	0.055 ^1^	12.2/17.1	0.320	3.2/7.4	0.195	3.2/17.1	**0.006**	7.4/17.1	0.066 ^1^
36 m	19.5/5.5	**0.007**	19.5/7.8	**0.001**	19.5/29.2	0.215	5.5/7.8	0.742	5.5/29.2	**0.003**	7.8/29.2	**0.005**

Bold: significant; ^1^ trend.

**Table 8:** Differences of mean values for “gap formation” (*p_i_*) within the groups over the period from BL to 36 months.

Parameter	Times	SBU-SE	SBU-SEE	SBU-ER	OFL
gap	BL vs. 6 m	**0.016**	0.125	0.063 ^1^	**0.008**
	BL vs. 12 m	**0.008**	0.063 ^1^	**0.016**	**0.008**
	BL vs. 24 m	**0.008**	0.063 ^1^	**0.016**	**0.004**
	BL vs. 36 m	**0.001**	**0.031**	**0.016**	**0.002**
	6 m vs. 12 m	1.000	0.844	**0.016**	0.063 ^1^
	6 m vs. 24 m	**0.023**	0.438	**0.016**	**0.004**
	6 m vs. 36 m	**0.001**	0.297	0.078	**0.002**
	12 m vs. 24 m	**0.008**	0.313	0.297	**0.004**
	12 m vs. 36 m	**0.001**	0.156	0.547	**0.002**
	24 m vs. 36 m	**0.001**	0.094	0.578	**0.002**

Bold: significant; ^1^ trend.

## Data Availability

Data supporting reported results are given in the respective tables.
